# Of Mice, Cattle, and Men: A Review of the Eco-Epidemiology of *Leptospira borgpetersenii* Serovar Ballum

**DOI:** 10.3390/tropicalmed6040189

**Published:** 2021-10-20

**Authors:** Marie Moinet, David A. Wilkinson, Danielle Aberdein, James C. Russell, Emilie Vallée, Julie M. Collins-Emerson, Cord Heuer, Jackie Benschop

**Affiliations:** 1 School of Veterinary Science, Massey University, Palmerston North 4442, New Zealand; D.A.Wilkinson@massey.ac.nz (D.A.W.); D.Aberdein@massey.ac.nz (D.A.); E.Vallee@massey.ac.nz (E.V.); J.M.Collins-Emerson@massey.ac.nz (J.M.C.-E.); C.Heuer@massey.ac.nz (C.H.); j.benschop@massey.ac.nz (J.B.); 2 New Zealand Food Safety Science and Research Centre, Hopkirk Research Institute, Palmerston North 4442, New Zealand; 3 Department of Statistics, School of Biological Sciences, University of Auckland, Auckland 1010, New Zealand; j.russell@auckland.ac.nz

**Keywords:** disease ecology, emerging infectious diseases, infectious disease reservoirs, liaison host, species barrier, wildlife–livestock interface

## Abstract

In New Zealand (NZ), leptospirosis is a mostly occupational zoonosis, with >66% of the recently notified cases being farm or abattoir workers. Livestock species independently maintain *Leptospira borgpetersenii* serovar Hardjo and *L. interrogans* serovar Pomona, and both are included in livestock vaccines. The increasing importance in human cases of Ballum, a serovar associated with wildlife, suggests that wildlife may be an overlooked source of infection. Livestock could also act as bridge hosts for humans. Drawing from disease ecology frameworks, we chose five barriers to include in this review based on the hypothesis that cattle act as bridge hosts for Ballum. Using a narrative methodology, we collated published studies pertaining to (a) the distribution and abundance of potential wild maintenance hosts of Ballum, (b) the infection dynamics (prevalence and pathogenesis) in those same hosts, (c) Ballum shedding and survival in the environment, (d) the exposure and competency of cattle as a potential bridge host, and (e) exposure for humans as a target host of Ballum. Mice (*Mus musculus*), rats (*Rattus rattus*, *R. norvegicus*) and hedgehogs (*Erinaceus europaeus*) were suspected as maintenance hosts of Ballum in NZ in studies conducted in the 1970s–1980s. These introduced species are distributed throughout NZ, and are present on pastures. The role of other wildlife in Ballum (and more broadly *Leptospira*) transmission remains poorly defined, and has not been thoroughly investigated in NZ. The experimental and natural Ballum infection of cattle suggest a low pathogenicity and the possibility of shedding. The seroprevalence in cattle appears higher in recent serosurveys (3 to 14%) compared with studies from the 1970s (0 to 3%). This review identifies gaps in the knowledge of Ballum, and highlights cattle as a potential spillover host. Further studies are required to ascertain the role that wild and domestic species may play in the eco-epidemiology of Ballum in order to understand its survival in the environment, and to inform control strategies.

## 1. Introduction

*Leptospira* are conjectured to be saprophytic soil bacteria that evolved into pathogenic strains by adaptation to mammalian hosts, and genomic tools are beginning to unravel the diversity of the species in this genus [1,2]. More than 300 *Leptospira* serovars and 65 species or candidate species have been described internationally. Only eight serovars from five serogroups and two species have been isolated in New Zealand (NZ), namely *Leptospira borgpetersenii* serovars (sv) Hardjobovis, Ballum, Balcanica and Tarassovi, and *L. interrogans* sv Pomona, Copenhageni, Australis and Canicola. Australis and Canicola have been isolated from humans only [3].

In NZ, leptospirosis was initially named “dairy farm fever” or “swineherd’s disease” [4,5]. It remains, nowadays, closely associated with agricultural occupations, with around two-thirds of cases being abattoir or farmworkers [6]. In the 1970s, Hardjobovis and Pomona represented 99% of the notified human cases [7], and 80–90% of these were dairy farmworkers [8,9]. Livestock species were determined as maintenance hosts for those serovars. As observed by Hathaway [10] in the same NZ farm environment, cattle and pigs harboured Hardjobovis or Pomona, the brush-tailed possum (*Trichosurus vulpecula*) harboured sv Balcanica, and rodents and hedgehogs (*Erinaceus europaeus*) harboured sv Ballum. Titres in livestock other than to Hardjobovis or Pomona were attributed to cross-reactivity and spillover events between wild and domestic hosts, and were considered as sporadic [10,11]. In order to describe this host specificity, the terms ‘nidality’ and ‘ecological niche’ have been used [12,13,14]. In contrast to numerous other countries, rodents and wildlife have since been considered of minor importance in human leptospirosis in NZ [10,15].

While human cases attributed to sv Ballum (i.e., Ballum hereinafter unless indicated otherwise) represented less than 1% of notifications in the 1970s–1980s [7], they now represent up to one-third of all cases [6,16,17]. Although more sensitive PCR tests are now available, serovar determination still relies on serological tests, for which the methodology has remained similar since the 1970s, and the evolution of laboratory diagnostics cannot explain this change. This apparent increase is relative and can, at least partially, be explained by the decreasing incidence of Hardjobovis and Pomona cases since the introduction and uptake of vaccination in the dairy and pig industries [18]. However, the absolute number of Ballum cases has also increased: the incidence of cases attributed to Ballum, which was negligible in the 1970s, increased from 0.2/100,000 in 1990–92 to 0.6/100,000 in 1996–1998 [19]. Nisa et al. [20] reported a 1.59-fold increase (95% CI 1.22–2.09) of the incidence in 2008–2017 (0.38/100,000) compared to 1999–2007 (0.23/100,000) for this serovar. In 2017, the Ballum incidence was the highest ever recorded in NZ, at 0.77/100,000 inhabitants [17].

This increasing importance of Ballum raises the need to better understand the eco-epidemiology of this emerging serovar. Wildlife may be an overlooked source of infection for humans. Farmers are among the notified Ballum cases, though the role of livestock in the eco-epidemiology of this serovar is unclear [21], and they could act as bridge hosts. This article reviewed literature on the eco-epidemiology of *L. borgpetersenii* sv Ballum relevant to NZ, and explored the possibility for this serovar to spill over into domestic hosts.

## 2. Methodology and Scope of This Review

The online databases used were Scopus, Web of Science, and SciQuest. Google Scholar, the Massey University library database (Discover) and NZResearch.org.nz were searched for additional grey literature on *Leptospira* in NZ, as well as the paper archives of the Leptospirosis Reference Centre in Amsterdam (spanning 1915–1990).

**Figure 1:**
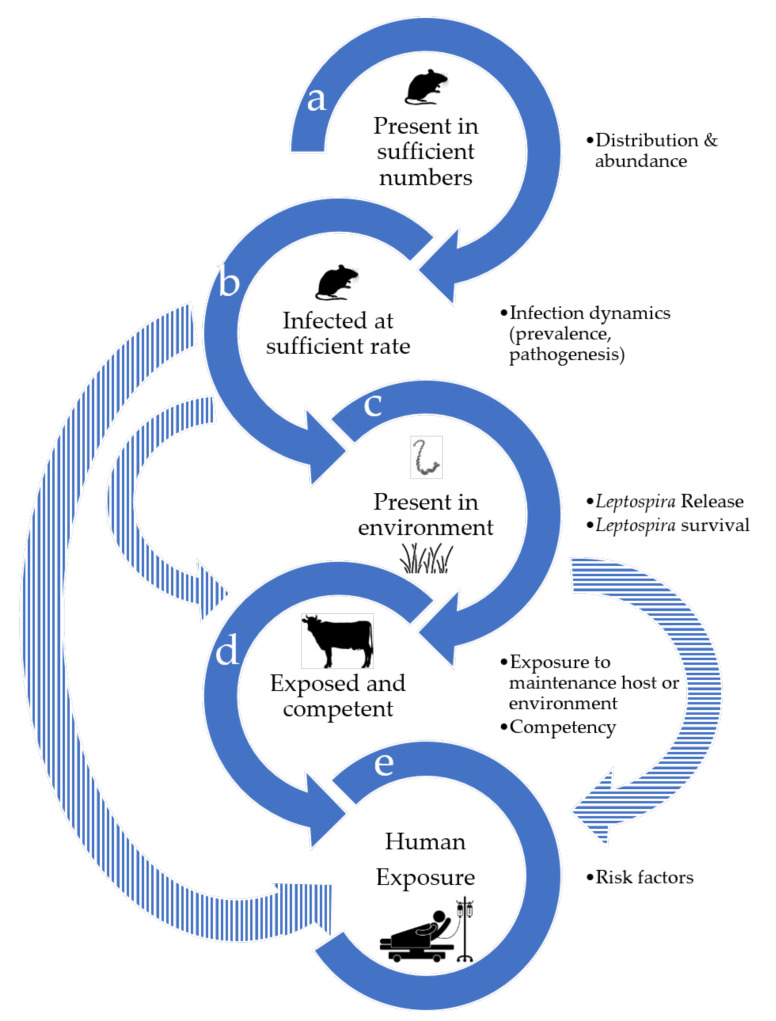
Theoretical barriers to Ballum spillover from wildlife to humans included in the literature search. This diagram assumes that wild mammals are maintenance hosts, cattle act as bridge hosts, and humans are target hosts. The plain arrows indicate the longest chain of transmission leading to spillover, while the striped arrows show possible shortcuts. The bullet points detail what is included in each barrier.

We used a narrative approach to conduct this review. First, we reviewed the history of Ballum worldwide and in NZ. Then, a preliminary literature search on wildlife as a source of *Leptospira* infection in NZ was performed (Appendix A and Supplementary Materials S1), informing a list of maintenance hosts to include in the rest of this review (barriers a and b; see below): the house mouse (*Mus musculus*), ship rat (*Rattus rattus*), brown rat (*R. norvegicus*) and hedgehog. Finally, drawing from two disease ecology frameworks conceptualising the barriers to be crossed for pathogen spillover [22] and the notion of bridge host [23], we chose five barriers (a–e) to include in the review based on the hypothesis that cattle act as bridge hosts (Figure 1):

(a) the presence and abundance of maintenance hosts;
(b) the infection dynamics of Ballum in maintenance hosts, including its prevalence and pathogenesis;
(c) Ballum shedding and survival in the environment;
(d) the exposure and competency of cattle as a potential bridge species; and
(e) the exposure (risk factors) for humans (target hosts).

The genetic, physiological and immunological attributes of the spillover hosts, while important determinants of transmission [22], were considered to be beyond the scope of this work. As meat workers had a lower incidence than farmers for Ballum [20], and as dairy farmers are in more frequent contact with livestock species compared to other farmers, we limited the notion of bridge host to this production system.

## 3. First Descriptions of Ballum

The type strain for serovar Ballum (Mus 127) was first isolated from a house mouse on a Danish farm in 1943 [24]. Another strain (S-102) had previously been isolated from a laboratory white mouse in Amsterdam in 1941, but wartime conditions limited further investigations [25].

The serogroup Ballum includes *L. borgpetersenii* svs Ballum, Castellonis, Arborea, Kenya, Guangdong and Soccoestomes, as well as *L. santarosai* sv Peru, and the more recently described *L. mayottensis* sv Kenya [26,27,28]. Standard serological methods like the microscopic agglutination test (MAT) are not serovar- but rather serogroup-specific, which limits the interpretation of serological data in areas where several of those serovars can be present.

Worldwide, Ballum has been isolated in a variety of locations and hosts, often rodents of the Muridae or Cricetidae families (e.g., [29]), but also Didelphidae (common and Virginia opossums *Didelphis marsupialis* and *D. virginiana*), various carnivores [30] and snakes (hog-nosed snake *Heterodon platyrhinus*) [14,31]. In human cases, this serovar is common in Europe and the Americas, but is less frequently reported in Asia [32]. Wild or laboratory house mice were proposed as the main reservoir of this serovar [33,34], probably because of their cosmopolitan and commensal nature, and because of the frequency of isolation of Ballum from this species. Isolation was occasionally successful from brown and ship rats, for instance in Portugal [35], Britain [36], Puerto Rico, British Columbia [31], Hawaii [37] and Italy [38], but they are more commonly associated with serovars from the Icterohaemorrhagiae serogroup [33,34]. Hedgehogs are more commonly associated with serovars within the Australis serogroup in Europe [39,40], but one strain of Ballum, Kipod 88, was isolated from the kidneys and brain of a hedgehog in an urban area in Israel (Tel-Aviv) in 1957 [41].

Early serosurveys conducted on *Leptospira* in animals and humans in NZ did not include this new serogroup (Ballum) in their testing panel [42,43,44,45,46,47,48]; see also Appendix A and Supplementary Materials S1. Smith was the first, in 1963, to include it in the initial screening phase of his macroscopic agglutination test panel, pooled with Canicola and Icterohaemorrhagiae [49]. Of the 98 hedgehog sera sampled in Hamilton, Upper Hutt, Dunedin, and Auckland, three were reactive to this antigen pool, all from the Hamilton area, and two failed to agglutinate with the specific Icterohaemorrhagiae and Canicola antigens. Smith did not have Ballum-specific antigens available for further investigation, and only attempted a culture on a small number of animals. Eight guinea pigs were inoculated with a mix of kidney and urine from 12 hedgehogs from the Hamilton area. Although two of the eight died, “apparently of leptospirosis”, he did not isolate any *Leptospira* from their blood, urine or kidneys, nor from the urine cultures of 16 additional hedgehogs ([49], p. 105). These results were not sufficiently reliable to ascertain the presence of Ballum in NZ.

The first NZ isolation of Ballum was performed in 1967 from two sick dairy farmworkers (Till (1968) in Ref. [11]), and from the urine of healthy calves six years later [50]. In another study, Ballum was included in the MAT panel, and all 26 possum sera tested were negative for this serovar [51]. It was not until 1976 [52] that the first isolations from wildlife were reported, from a brown rat and a rabbit (*Oryctolagus cuniculus*) trapped on an artificial breeding centre for cattle. The serotyping method was not detailed in the article, and only two brown rats, 11 rabbits, two possums, four hedgehogs and three hares were examined. Bovine urine (n = 102) and ovine kidneys (n = 28) were also sampled, and *Leptospira*-like organisms were seen using dark-field microscopy (DFM) in, respectively, 34 and five of them. However, the isolation—and therefore serotyping—of those organisms was not successful.

Most of the available information on Ballum infection in NZ wildlife comes from two concomitant research projects conducted in the late 1970s [10,53,54]. The first project, led by Brockie, initially focused on hedgehogs; the isolation of the serovar Ballum from the kidneys of five healthy female hedgehogs caught in dairy farms throughout the North Island led him to suggest that this species was a major reservoir of Ballum in NZ [54]. Brockie also trapped mice and brown rats harbouring Ballum in refuse dumps in the North Island [53]. The second project, carried out by Hathaway and supervised by Blackmore and Marshall, focused on possums and Balcanica [55,56], but they also conducted a survey investigating *Leptospira* in several species [57], and another focused on ship rats and brown rats in refuse dumps and farming environments [10]. They found that, contrary to ship rats, the Ballum culture prevalence was density-dependent for brown rats. It was concluded that ship rats were maintenance hosts, while brown rats were able to maintain Ballum only in high-density populations [58]. However, the study did not ascertain the presence or absence of mice in the refuse dumps where those rats were shot, and mice could have acted as the primary maintenance host for this serovar.

## 4. Barriers for Ballum Spillover

*(a) Maintenance Host Distribution*

Except brown rats, all of the recognised maintenance hosts of Ballum are distributed throughout NZ. Brown rats, mice and ship rats arrived in NZ with the first European settlers in the 1770s–1790s, 1790s–1840s and 1860s–1890s respectively [59,60], and rapidly colonized both the North and South Island. Brown rat populations that were thriving in the 1850s declined by the end of the 19th century, and have since had a discontinuous distribution that has been attributed to competition with ship rats and/or predation from mustelids released as an attempt to control rodents and rabbits [60,61].

Hedgehogs were first released around Christchurch and Dunedin in the 1870s–1890s, and had reportedly dispersed through most lowland areas of the South Island by 1910, when they were introduced and quickly spread in the North Island [62]. They are now present throughout NZ, except at high altitudes, and are found in gardens and urban areas, and also grassland and shrubland. In their native range, they are known to avoid pastures because of the risk of predation by badgers [63]. Comparisons of road-kill counts along North Island highways indicate that the abundance of hedgehogs was similar in 1984 and 1994, dropped drastically (−82%) in 2005 [64], and subsequently recovered between 2009 and 2014 [65].

The preferred habitat for mammal species introduced to NZ can differ from their original habitat in their native range. Mice, known to be commensal and found only around human dwellings or farm buildings, benefit from the absence of other wild rodents, and are also present in pastures and forests throughout NZ. The same is true for ship rats, which benefit from the absence of other arboreal rodents (such as squirrels) and are also present in forests [66]. Brown rats remained the most synanthropic. They are found more readily around dwellings, in suburbs and refuse dumps. They can be found in farm environments, but in lower densities than ship rats, and preferentially around farm buildings [61].

Almost all of the available density or abundance estimates published for rodents since 2004 were carried out in forests or islands in the frame of conservation projects (Table 1). Rodent density estimates in studies published before 2004 are listed in [67,68]. Populations are known to fluctuate greatly, with spikes associated with seed masting events [69,70]. Although mice and rats are known to be present in pastures, there is a dearth of information on their abundance in farm habitats. A study in grazed or fenced fragments of native forest showed a higher density of ship rats in the fenced fragments, and the highest measured densities in mainland NZ [71]. Mice benefit from the removal of predators [72] and rats [73], with which they compete, and this effect was expected to be especially true in warmer forests of NZ [70].

One study in Tāwharanui Open Sanctuary (Northland) compared the mouse relative abundance in forest, grazing pasture, coastal vegetation and rank grass before and after the removal of other invasive species. While undetected in pastures before removal, there were up to 3.5 mice captured per 100 trap-nights (C/100TN) after removal. This was significantly less than estimates in the three other habitats, which were between 120 and 190 C/100TN in the same period [72]. Another study described higher mice presence indices in former pastures which were being regenerated than in the neighbouring grazed pastures [74]. In forests, the relative abundance ranged between 0 and 62 mice C/100TN, and up to 190 C/100TN in the absence of competitors (Table 1). 

**Table 1:** Indices of relative abundance (in captures or corrected captures/100 trap-nights, or * in sighting/100 km) and density estimates (individuals per hectare) published in the literature for mice (*Mus musculus*), rats (*Rattus rattus*) and hedgehogs (*Erinaceus europaeus*) in New Zealand. The table was adapted and completed from [68] with the kind permission of the authors. Only literature published after 2004 was searched for rodents.

Place	Is.	Habitat Type	Abundance	Density	Months	Years	Ref.
*Mus musculus*							
Grebe Valley	SI	Beech forest	0.5–32.9	\	Feb, Dec	2000	[75]
Borland Valley	SI	Beech forest	0–62	\	Nov, Feb, Dec	1999–2000	[75]
Borland Valley	SI	Beech forest	\	0.02–1.8	Feb, May, Nov	2003–2004	[76]
Waitutu Forest	SI	Beech—mixed forest	\	8–28	F, M, A, N ^1^	2001–2003	[77]
Ōrongorongo Valley	NI	Beech—mixed forest	0–13.8	\	F, M, A, N ^1^	1973–1998	[78]
Maungatautari	NI	Podocarp—mixed forest †	\	9–46	F, M, A, N ^1^	2011–2016	[79]
Moturekareka Is.	oiNI	Coastal forest/scrub †	\	81	Apr	2014	[80]
Saddle Is.	oiNI	Coastal forest/scrub †	\	8.8–19.2	Jan, Mar, May, Aug	2008	[81]
Tawharanui	NI	Coastal forest/scrub †	1–190.16	14.6–156.7	F, A, J, A, O, D ^2^	2007	[72]
Maud Is.	oiSI	Coastal forest/scrub †	\	138	Feb	2014	[80]
Moturekareka Is.	oiNI	Pine forest †	\	34	Apr	2014	[80]
Auckland Is.	OI	Rata forest (+ shrubland)	5.6–7.2	\	Jun, Jul	2007	[82]
Maud Is.	oiSI	Scrub (Manuka/Grass) †	\	102	Feb	2014	[80]
Antipodes Is.	OI	Tussock/Grassland †	\	55–104	Jan, Jul	2011 and 2013	[83,84]
Borland Valley	SI	Tussock/Grassland	\	0.4–38.6	Feb/Mar, May, Nov	2003–2007	[76]
Auckland Is.	OI	Tussock/Grassland	12.7	\	Jun, Jul	2007	[82]
Tāwharanui	NI	Grassland (grazed pasture) †	0–3.51	\	F, A, J, A, O, D ^2^	2007	[72]
Tāwharanui	NI	Grassland (rank grass) †	1.71–121.13	\	F, A, J, A, O, D ^2^	2007	[72]
Waikauri Bay	NI	Grassland (rank grass)	17.62–91.18	\	Apr, Aug, Dec	2007	[72]
Tāwharanui	NI	Supra-littoral vegetation †	40–130.44	\	F, A, J, A, O, D ^2^	2007	[72]
*Rattus rattus*							
Eglinton Valley	SI	Beech forest	\	0.38	Mar	2005	[85]
Ōrongorongo Valley	NI	Beech—mixed forest	2.3–7.5	\	F, M, A, N ^1^	1971–1998	[86]
Ōrongorongo Valley	NI	Beech—mixed forest	31	5–9	Apr, May	2003–2004	[87]
Waikato	NI	Broadleaved forest fragment (fenced)	\	6.5	Jan, Feb	2008	[71]
Waikato	NI	Broadleaved forest fragment (grazed)	\	0.5	Jan, Feb	2008	[71]
Big South Cape Is.	oiStI	Supra-littoral vegetation	\	6.5–36.4	Dec, Jan	2003–2004	[67]
*Erinaceus europaeus*							
\	NI	Roadkill	0–58.3 *	\	Jan, Feb	1987	[88]
\	SI	Roadkill	0–8 *	\	Jan, Feb	1987	[88]
\	NI	Roadkill	6.7–6.9 *(max 23–25 *)	\	Feb	1984–1994	[64]
\	NI	Roadkill	1.3 *	\	Feb	2005	[64]
\	NI	Roadkill	4–25 *	\	\	2009–2014	[65]
Macraes flat	SI	Tussock/Grassland	0.01	\	May	2013	[89]
Tasman Valley	SI	Tussock/Grassland (shrubs)	0	\	Jun, Jul	2013	[89]
Lake Wairarapa	NI	Grassland/Scrub	\	0.88	Oct to May	1995–1996	[90]
Massey University	NI	Farmland	\	2.5	Nov to Jun	1970–1971	[91]
Massey University	NI	Farmland	\	1.1	Jul to Oct	1970–1971	[91]

Is. = Island, NI = North Island, SI = South Island, StI = Stewart Island, oiSI/NI/StI = outlying island of SI/NI/StI, OI = Offshore island; † Mice were the only non-native mammal species present; ^1^ Quarterly trapping: February, May, August and November; ^2^ Bi-monthly trapping: February, April, June, August, October, December.

*(b) Infection Dynamics in Maintenance Hosts*

(b1) Ballum Prevalence

The overall seroprevalence for Ballum described in the literature varied between 3 and 8% for mice [53,57], 6 and 28% for ship rats [53,57,58,92], 4 and 29% for brown rats [53,57,58,92,93] and 2 and 36% for hedgehogs [49,54,57], with a variable cut-off across the studies (from 12 to 100). The culture prevalence varied between 13 and 16% [53,57], 0 and 33% [53,57,58,92,93], and 0 and 19% [49,54,57,94], respectively (Table 2). No PCR methods were used except for two pilot studies carried out prior to this work on a small number of animals (reporting a total of 3/7, 0/2, and 0/1 PCR positive hedgehogs, mice and “rats”, with a PCR targeting the 16s rRNA gene) [95,96]. Some studies did not allow the distinction between ship and brown rats [93,96,97]. Other studies had a sample size too low to allow for an estimation of prevalence [52,95,96,97]. There was no estimate available after 1999.

Both Hathaway and Brockie found that among rodents with a Ballum-positive culture, the majority were seronegative, and that serology was not reliable for the diagnosis of the infection status of these animals. In total, 46 to 89% of brown rats, 67 to 75% of ship rats, and 83 to 89% of mice with a Ballum isolate were seronegative [53,57]. Brockie also noted that one in five hedgehogs shedding Ballum had no detectable antibodies [53].

A statistically significant difference (Fisher’s exact test *p*-value = 0.0259) was reported between hedgehogs in urban and farm environments despite the small sample size of the urban hedgehogs. Six urban hedgehogs showed no seropositive reactions and no isolation, while 56% of the 72 hedgehogs captured on dairy farms had evidence of infection [54], which was in agreement with Smith’s results in 98 urban hedgehogs, with only two being seropositive for Ballum (pooled with Canicola and Icterohaemorrhagiae) [49]. However, in a recent pilot study, Ballum was isolated from one of five urban hedgehogs [96].

A lower prevalence was also described in urban rodents compared to those in rural habitats, again relying on low numbers of urban animals (respectively 0/4, 0/2 and 0/3 urban mice, brown rats and ship rats vs. 10/73, 19/76 and 4/14 rural mice, brown rats and ship rats showed culture or serological evidence of infection with *Leptospira* sp.) [53,54]. However, contrasting results were also described, with a significantly higher prevalence of Ballum and Copenhageni in brown rats in urban habitats (11/12 Ballum isolates from urban brown rats), while ship rats had a higher prevalence in rural areas (one of three Ballum isolates from urban ship rats) [93]. In this study, the data were presented with both rat species pooled, and the exact number of urban/rural rats of each species sampled—and hence the prevalence—could not be inferred.

All of the NZ studies available in the literature investigating the seroprevalence and prevalence of Ballum are cross-sectional surveys, and therefore single time-point estimates. No work was conducted on the dynamics of the infection in these populations. A study from New Caledonia investigated the dynamics of rodents and *Leptospira* carriage over time, and linked a higher prevalence to hot and rainy seasons [98]. Despite Ballum being putatively identified (congruent Multi-Locus Sequence Type) in ship rats and mice, these results cannot be extrapolated to NZ, where the climate is different.

**Table 2:** Summary of the published studies investigating *Leptospira borgpetersenii* serovar Ballum in wild species in New Zealand. For each study and species (Sp) are indicated: the numbers of seropositive animals (Sero + ve) and animals tested by serology (#S), the seroprevalence (Seroprev), the numbers of culture-positive animals (Cult + ve) and animals tested by culture (#C), and the culture prevalence (Cult prev). An extended version of this table with information on other serovars and studies not testing for Ballum [42,43,44,45,46,47,48,56,99,100,101,102,103,104,105,106,107] is available in the Supplementary Materials S1.

Sp ^‡^	α	Place	Habitat ^†^	Test ^§^	Test Cut-off	Sero +ve	#S	Sero prev	Cult +ve	#C	Cult prev	Reference
Ee	κ	Hamilton, Upper Hutt, Dunedin, Auckland	Urb, Suburb	AT	\	3	98	3%	0	28	0%	[49]
Ee	κ	NZ–NS	Urb, Suburb	NS	NS	.	98	\	0	11	0%	[94]
Ee	κ	Bulls, Manawatū	Rural	MAT	20	0	4	0%	0	4	0%	[52]
Ee	κ	North Island	Farm	MAT	100	28	78	36%	5	78	6%	[54]
Ee	κ	NZ–NS	Rural	MAT	100	0	1	0%	0	1	0%	[97]
Ee	κ	North Island	Farm, Forest, Urb	MAT	24	9	25	36%	5	27	19%	[57]
Ee	π	North Island	Rural	MAT	48	1	2	50%	0	2	0%	[95]
Ee	π	Palmerston North	Urb, Suburb	MAT	48	1	5	20%	1	5	20%	[96]
Tv	κ	Whanganui district	Farm	MAT	200	0	26	0%	0	NS	0%	[51]
Tv	κ	Bulls, Manawatū	Rural	MAT	20	0	2	0%	0	2	0%	[52]
Tv	κ	North Island	Farm	MAT	24	\	127	\	\	\	\	[55]
Tv	κ	North Island	Farm, Forest, Urb	MAT	24	11	754	1%	0	27	0%	[57]
Tv	κ	Ōrongorongo valley	Forest	MAT	24	1	261	0.40%	0	247	0%	[108]
Tv	π	North Island	Rural	MAT	48	0	21	0%	0	1	\	[95]
Tv	π	Palmerston North	Urb, Suburb	MAT	48	2	16	13%	0	26	0%	[96]
Rn	κ	Bulls, Manawatū	Rural	MAT	20	1	2	50%	1	2	50%	[52]
Rn	κ	North Island	Urb, Rural	MAT	100	7	79	9%	8	79	10%	[53]
Rn	κ	Waikato	Urb, Rural	MAT	20	26	134	19%	12	132	9%	[93]
Rn	κ	North Island	Farm, Forest, Urb	MAT	24	6	168	4%	63	245	26%	[57]
Rn	κ	Manawatū	Farm, Forest	MAT	12	6	168	4%	63	243	26%	[58]
Rn	κ	North Island	Farm, Urb	MAT	24	2	7	29%	0	7	0%	[92]
Rn	π	North Island	Rural	MAT	48	1	1	100%	\	\	\	[95]
Rr	κ	North Island	Urb, Rural	MAT	100	1	16	6%	4	16	25%	[53]
Rr	κ	Waikato	Urb, Rural	MAT	20		.	.	3	28	11%	[93]
Rr	κ	North Island	Farm, Forest, Urb	MAT	24	8	29	28%	21	63	33%	[57]
Rr	κ	Manawatū	Farm, Forest	MAT	12	8	30	27%	21	61	34%	[58]
Rr	κ	North Island	Farm, Urb	MAT	24	4	17	23%	0	17	0%	[92]
“Rat”	κ	NZ–NS	Rural	MAT	100	0	15	0%	0	15	0%	[97]
“Rat”	π	Palmerston North	Urb, Suburb	MAT	48	\	0	\	\	\	\	[96]
Mm	κ	North Island	Urb, Rural	MAT	100	2	67	3%	9	67	13%	[53]
Mm	κ	North Island	Farm, Forest, Urb	MAT	24	3	39	8%	11	70	16%	[57]
Mm	π	North Island	Rural	MAT	48	0	1	0%	0	1	0%	[95]
Mm	π	Palmerston North	Urb, Suburb	MAT	48	\	0	\	\	\	\	[96]
Af	λ	Otago Peninsula	Seashore	MAT	100	0	128	0%	\	\	\	[109]
An	κ	North Island	Farm, Forest, Urb	MAT	24	0	29	0%	0	29	0%	[57]
As	κ	North Island	Farm, Forest, Urb	MAT	24	3	29	10%	0	29	0%	[57]
Ce	κ	North Island	Farm, Forest, Urb	MAT	24	0	27	0%	0	3	0%	[57]
Ch	κ	Raglan county	Rural	MAT	24	4	116	3%	0	101	0%	[110]
Cn	κ	North Island	Farm, Forest, Urb	MAT	24	0	4	0%	0	2	0%	[57]
“Deer”	κ	NZ–NS	NS	NS	NS	0	15	0%	\	\	\	[94]
Fc	κ	North Island	Farm, Suburb	MAT	24	1	11	9%	0	11	0%	[15]
Fc	π	North Island	Rural	MAT	48	0	14	0%	0	3	0%	[95]
Le	κ	Bulls, Manawatū	Rural	MAT	20	0	3	0%	0	3	0%	[52]
Le	κ	North Island	Farm, Forest, Urb	MAT	24	0	5	0%	0	5	0%	[57]
Me	κ	North Island	Farm	MAT	24	0	9	0%	0	9	0%	[15]
Me	π	North Island	Rural	MAT	48	0	1	0%	\	\	\	[95]
Me	π	Palmerston North	Urb, Suburb	MAT	48	0	2	0%	0	2	0%	[96]
Me*	π	Rotorua area	Forest	MAT	NS	4	39	10%	0	39	0%	[111]
Mf	κ	North Island	Farm	MAT	24	0	9	0%	0	9	0%	[15]
Mf	π	North Island	Rural	MAT	48	0	1	0%	\	\	\	[95]
Mn	κ	North Island	Farm	MAT	24	0	4	0%	0	4	0%	[15]
Mn	π	North Island	Rural	MAT	48	\	\	\	\	\	\	[95]
Oc	κ	Bulls, Manawatū	Rural	MAT	20	1	11	9%	1	11	9%	[52]
Oc	κ	North Island	Farm, Forest, Urb	MAT	24	0	9	0%	0	9	0%	[57]
Oc	π	North Island	Rural	MAT	48	0	1	0%	0	1	0%	[95]
Pm	κ	North Island	Farm, Forest, Urb	MAT	24	0	34	0%	\	0	\	[57]
Ss	π	North Island	NS	MAT	NS	0	60	0%	0	39	0%	[111]

^‡^ Species: Af = NZ fur seal, *Arctocephalus forsteri*; An = Mallard duck, *Anas platyrynchos*; As = Grey duck, *Anas superciliosa;* Ce = Red deer, *Cervus elaphus*; Ch = Feral goat, *Capra hircus*; Cn = Sika deer, *Cervus nippon*; “Deer” = species not indicated; Ee = European hedgehog, *Erinaceus europaeus*; Fc = Feral cat, *Felis catus*; Le = European brown hare, *Lepus europaeus*; Me = Stoat, *Mustela erminea*; Me* = not specified but likely Dama wallaby, *Macropus eugenii*; Mf = Ferret, *Mustela putorius furo*; Mm = House mouse, *Mus musculus*; Mn = Weasel, *Mustela nivalis*; Oc = European rabbit, *Oryctolagus cuniculus*; Pm = Pukeko, *Porphyrio melanotus*; “Rat” = species not indicated (could be Rn or Rr); Rn = Brown rat, *Rattus norvegicus*; Rr = Ship rat, *Rattus rattus*; Ss = Feral pig, *Sus scrofa*; Tv = Brushtail possum, *Trichosurus vulpecula*; NS: not specified/not applicable; α, type of study: κ = cross-sectional survey, λ = longitudinal survey, π = pilot study; ^†^ Urb = Urban, Suburb = Suburban (defined according to its original meaning as areas in the periphery of urban centres, this category includes refuse dumps); ^§^ MAT = Microscopic Agglutination Test, AT = Agglutination lysis test.

(b2) Pathogenesis

The literature on the pathogenesis of *Leptospira* in laboratory animals is abundant, especially on mice that are widely used as models of sublethal infection [112], but descriptions of natural infections in wild populations are scarce [113]. Infection is asymptomatic for mice and rats, and no experimental study on hedgehogs is available for Ballum. Hathaway determined a minimum infective dose of 10 bacteria for Ballum and Pomona in pathogen-free laboratory mice injected intraperitoneally [10,114]. This dose was much higher (10^7^ organisms) for Balcanica and Hardjo.

Experimental infections with a fatal outcome were described for hedgehogs infected with Pomona [47] and a combination of Szwajizak and Canicola (by instillation into the nostrils) [41]. The natural route of infection for hedgehogs is thought to be by direct contact between nasal and/or buccal mucosa and contaminated water while foraging for food [54]. Fennestad and Borg-Petersen [115] described a positive correlation between interstitial nephritis and *Leptospira* infection in hedgehogs in Denmark, but the dominant serovar isolated from this population was Bratislava.

*(c) Pathogen Release and Survival in the Environment*

(c1) Pathogen Release

The amount of *L. borgpetersenii* sv Ballum released in the urine of maintenance hosts has not been studied in natural environments. For *Leptospira* spp., a recent meta-analysis [116] gave a quantitative estimate for different hosts, including rats (5.7 × 10^6^ *Leptospira*/mℓ of urine), mice (3.1 × 10^3^ *Leptospira*/mℓ), cattle (3.7 × 10^4^ *Leptospira*/mℓ) and humans (7.9 × 10^2^ *Leptospira*/mℓ). However, the low number of subjects, the variation in the methods used to quantify the bacterial load between studies, and the fact that different species and serovars of *Leptospira* were considered, limit the possible comparisons between host species. The presence and amount of *Leptospira* in the kidney of a host is often inferred to directly reflect the presence and amount of *Leptospira* in voided urine. Using quantitative PCR, Costa et al. [117] indeed found a significant positive correlation between the average load of *Leptospira* in the kidneys and urine samples of brown rats. Leptospiruria has, however, been described as being intermittent, of variable length both within and between species, and also dependent on the infecting strain, but this may be due to the use of insensitive methods like DFM [10]. Reviews mentioning intermittent shedding refer to previous studies conducted without PCR methods (e.g., [113] quoted [118], who used culture and DFM, [119] quoted [120]) and it is probable that intermittent excretion was in fact intermittent detection. Experimental infections with Ballum in mice showed that, after infection, they began shedding rapidly and reached a plateau of 3 × 10^7^ *Leptospira*/mℓ of urine after 117 days, and kept shedding virtually until the end of their lives [121]. A positive association was described between the renal *Leptospira* load and weight in male rats [122].

(c2) Pathogen Survival, Development, and Dissemination Outside the Host

We found no study investigating the specific survival and development of Ballum in situ (i.e., in the environment). One study investigated Castellonis (strain Castellón 3), another serovar from the Ballum serogroup, and showed that, in vitro, with sterile conditions at pH 7.2, it survived 9 days at 4 °C, 32 days at ambient temperature, and 155 days at 30 °C [123]. The addition of saprophytic leptospires (*L. biflexa* sv Patoc 1) did not affect the survival and virulence of this serovar in guinea pigs [123]. Brockie and Till [54] hypothesized that because hedgehog urine was acidic, leptospires would not survive long in their urine unless they were directly voided in water, on pastures or soil.

The species *L. borgpetersenii* has a smaller genome than *L. interrogans*, and by comparing their genomes (two strains of sv Hardjobovis vs. svs Lai and Copenhageni), Bulach et al. [124] hypothesized that the former underwent a process of genome reduction, losing mainly genes which were important for its adaptation and survival in the environment. They linked this difference in genome size to a difference in the transmission process, with *L. borgpetersenii* sv Hardjo having a direct animal-to-animal transmission, rather than an indirect transmission through the environment. By comparing *L. borgpetersenii* with both *L. interrogans* and *L. biflexa*, Picardeau et al. [125] confirmed that the loss of transduction functions in *L. borgpetersenii* impacts its ability to survive outside its host. The absence of environmental transmission was confirmed experimentally for *L. borgpetersenii* sv Balcanica in possums, for which transmission is thought to occur during mating [104,105]. The survival of *L. borgpetersenii* sv Ballum in the environment should thus be, at least theoretically, limited.

The importance of abiotic factors like pH, humidity, temperature, salinity, and UV light for the survival of *Leptospira* spp. in the environment has long been recognized [126,127,128]. The physicochemical properties of soil also play a role in the survival of leptospires, but very little is known on this topic for leptospires in general, and even less so for Ballum. In Ontario, the distribution of pathogenic and saprophytic *Leptospira* serovars was correlated to the type of bedrock, with titres to pathogenic leptospires (mainly Pomona) being found only in animals from areas with Paleozoic bedrock, while titres to saprophytes (*L. biflexa*) were more ubiquitous [129]. Lall [130] described a significant positive relationship between the presence of *Leptospira* and soil concentrations of iron, manganese and copper. Soil is suspected to be a better habitat than water for *Leptospira* survival [131].

The capacity to form biofilms and resist harsh environmental conditions has been described in a variety of pathogenic and saprophytic strains of *Leptospira* spp., including *L. borgpetersenii* svs Castellonis, Hardjobovis, Sejroë and Tarassovi [132,133]. Again, no information on this trait has been published on Ballum, but its capacity to aggregate in cultures suggests that it can likely form biofilms as well.

Other bacteria present in the environment can interact with and decrease *Leptospira* survival: *Aerobacter cloacae* and *Pseudomonas* spp. (Abdoelrachman (1947) in [128]); or, on the contrary, increase it: *Escherichia coli, Mycobacterium rubra* (Abdoelrachman (1947) in [128]), *Sphingomonas* spp. [134], *Azospirillum* spp., *Micrococcus* spp., *Brevundimonas* spp., *Acinetobacter* spp., and *Paracoccus* spp. [135]. By forming biofilms with other bacteria, *Leptospira* were more resistant to ultraviolet light, temperature stress and antibiotics [134,135]. It is not known if different serovars react differently to the presence of other bacteria, and if these findings can be extrapolated to Ballum. More work is needed to understand the factors affecting the environmental survival of Ballum, and more broadly, of *Leptospira* spp. [131,136].

*(d) Exposure and Competency of Cattle*

Cattle appear to be a competent host of Ballum, and seem to be more exposed to Ballum in recent serosurveys compared to older ones. Ballum was first isolated in NZ from two healthy calves, during a study conducted on nine calf groups in the Hauraki Plains [50]. For the same study in asymptomatic calves, the 1972–1973 annual report of the Wallaceville Animal Research Centre mentioned the presence of anti-Ballum antibodies in 44% of them (18/25 and 4/25 in two of the nine groups) [137]. The facts that the cut-off for seropositivity was not specified, and that the results were not detailed in the associated publication—only titres of leptospiruric calves were reported—call into question the reliability of this reported seroprevalence. In an experimental infection of Ballum in two 8 to 10-week-old calves [138], the temperature peaked three days post-infection (PI) and lasted no more than two days. Leptospiruria was observed between 24 and 68 days PI, and MAT titres of up to 10,000 were observed at 10 days PI. Complement fixation (CF) antibodies peaked at 10 days PI and declined more rapidly than MAT titres, being detectable for only 8 to 23 weeks PI [138]. Hodges [139] found no haemolytic effect of Ballum antigens on cattle erythrocytes. He suspected the presence of haemolysin inhibitors in convalescent sera from cattle infected with Ballum or Hardjobovis that only partially inhibited the haemolysis induced by Pomona antigens, and only at low dilutions. In comparison, the inhibition of haemolysis induced by Pomona antigens was complete for convalescent sera from cattle infected with Pomona or Copenhageni [139]. Thus, the pathogenicity of Ballum (and Hardjobovis) appeared to be lower than that of other highly pathogenic serotypes.

Despite no evidence of the pathogenicity of Ballum in cattle in peer-reviewed publications, there were clinical cases described in the grey literature: severe clinical signs of photosensitisation attributed to Ballum were described in more than 17% of a mob of 3-month-old Friesian calves. Two calves died and showed severe subcutaneous oedema, skin necrosis and sloughing. Two other cases were sampled, and showed leptospiruria and “seroconversion to Ballum only” ([140], p. 12). The other serovars tested were not specified in the report. Another report described the case of a calf “doing poorly” that had “red-discoloured urine”, with analyses revealing a mild multifocal cortical interstitial nephritis, with a positive *Leptospira* PCR on urine, but no *Leptospira* visible by silver staining, and titres of 400 for Ballum but seronegative for Pomona and Hardjobovis [141]. Two of the ten asymptomatic calves subsequently tested in the same mob also had titres for Ballum (400 and 1600). These results must be interpreted with caution, as the involvement of Ballum here was based on only indirect evidence. However, in another report, Ballum was isolated from the liver and urine of a 4-week-old calf that died of leptospirosis [142]. This calf had haematuria and pale mucous membranes, and the report mentioned lesions of severe focal nephritis and “some haemoglobin casts” in the renal convoluted tubules [142]. The method used to type the Ballum isolate was not indicated.

A very small proportion (3/10,680 in 1973, 3/6409 in 1974, 9/1020 in 1977, 0/257 in 1978) of abortive cows routinely tested by serology at the Ruakura Animal Health Laboratory showed antibodies for Ballum (titres ≥ 200) [143,144,145,146]. The CF test, which was not serovar-specific, was introduced in 1978 to replace MAT for the routine diagnosis of leptospirosis [146], and information on the serovar was subsequently not available.

Among the descriptive and analytical studies on *Leptospira* infection in cattle in NZ published in the last 40 years, only the most recent included Ballum in their MAT antigenic panel [147,148]. The others only targeted Pomona and Hardjo [149,150,151,152,153,154,155].

**Figure 2:**
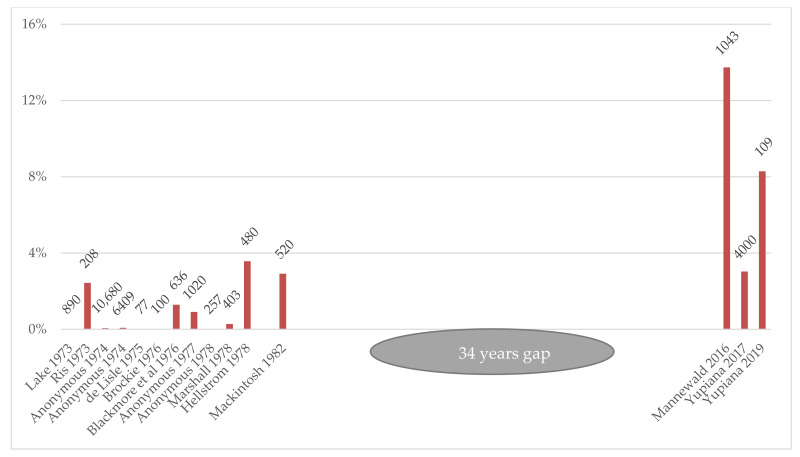
Crude seroprevalence of *L. borgpetersenii* serovar Ballum in cattle and numbers tested reported in the New Zealand literature since the first description of this serovar in the country.

The crude seroprevalence of Ballum (48 seropositivity threshold) was 13.7% (95% CI 11.7–16.0%) in beef cattle [147] and 3% (95% CI 3–4%) in dairy cattle [156], with at least one positive animal in 76% of beef herds and 38% of dairy herds. All of the titres described were <384. The animals sampled in those two studies were all adults, but as dairy cattle are usually kept longer than beef cattle, the average age of the animals tested likely differed. Although there was an apparent increase in Ballum seroprevalence between the 1970s and 2010s (Figure 2), the age at sampling appears to be an important factor, with the highest prevalence observed in calves. This makes comparisons of studies and the identification of a real increase difficult.

There is no study available on the Ballum seroprevalence dynamics in naturally infected cattle, or the duration of titres over time. However, the use of serology to assess exposure may be limited by the presence of seronegative carriers. Using a *gyraseB* PCR, Yupiana et al. [157] found 94/4000 urine samples of adult dairy cows to be positive, with 13/81 of amplicons subsequently sequence-typed as Ballum, all from cows which were seronegative for Ballum. This discrepancy between the MAT titres and culture or PCR results has also been identified with other species of *Leptospira* [158]. The seroprevalence presented in the previous studies cited above is therefore likely to represent an underestimation of the true prevalence.

The hypothesis of ‘competitive exclusion’ between serovars within a mammalian host has been proposed in the past [13]. Hathaway suggested the widespread use of vaccines against Hardjobovis and Pomona in cattle would create an empty ‘niche’ that could benefit other serovars. Vaccination against Hardjobovis and Pomona has been in place since the early 1980s in NZ. The coverage varies according to the farming type, with 99.5% of dairy cattle being vaccinated [157] compared with only around 5–25% of beef and deer, and less than 1% of sheep [159]. Yupiana et al. showed that vaccination and antibiotic treatments were efficacious in reducing the risk of shedding *Leptospira* spp. in dairy cows, but also reported evidence of some animals shedding Ballum after vaccination [160]. According to this ‘competitive exclusion’ hypothesis, the emergence of a new serovar in cattle populations would be more likely in dairy cattle than beef cattle, which contrasts with the recent seroprevalence estimates observed in those two groups.

*(e) Human Exposure and Risk Factors*

Most studies on human exposure have focused on high-risk occupations in the agricultural sector, and while Hardjobovis and Pomona cases are mostly related to farm and meat workers, infections with Ballum do not appear to share the same pattern. Leptospirosis is a notifiable disease in NZ, and is also covered as an occupational disease for farmers and meat industry workers [161]. There is probably a bias in notification towards these occupations, but even so, the total number of severe cases was estimated to be approximately three times higher than the notifications. When mild human leptospirosis cases—that are more likely to be missed—were taken in consideration, this under-ascertainment factor increased to 22-fold [162].

Among the 1999–2017 notified cases, the odds of Ballum infection were lower for meat workers compared to farmers (adjusted OR 0.05, 95% CI 0.02–0.13), but almost three times higher for ‘other’ occupations compared to farmers (adjusted OR 2.61, 95% CI 1.64–4.14), although farmers and meat workers showed a higher mean incidence for Ballum than the whole population [163]. People with Ballum infections were significantly older, and the incidence in people of European ethnicity was also significantly higher than in Māori, while the contrary was observed for Hardjobovis and Pomona [20,163]. Interestingly, the annual incidence for Ballum in stock farmers and dairy farmers was relatively similar to the incidence of Hardjo (the average incidence was, respectively, 13.1 and 7.3/100,000 for Ballum vs. 18.2 and 11.3/100,000 for Hardjo for the 2008–2017 period), but much lower in meat workers (2.0/100,000 compared to 38.7/100,000) [20]. Another study indicated that forestry-related workers were also at risk for Ballum, with 57.1% of cases being due to this serovar in this occupation group [19].

Among 302 veterinary students enrolled at Massey University, NZ, Fang et al. [164] found none who were reactive to *Leptospira* spp. using MAT and testing for Hardjobovis, Pomona and Ballum. Among 277 veterinarians, Sanhueza et al. [165] found only one with a titre of 48 for Ballum (0.4%, 95% CI 0–2.0%). Dreyfus et al. [166] did not test for Ballum in their study on meat workers. A study was conducted in 1974–1976 using the CF test, rather than the MAT, on the NZ National Serum Bank. Sera from 879 donors in Wellington and Christchurch, with occupations other than farmer or meat worker, had no detectable antibodies against *Leptospira* spp. [167].

Ballum and Pomona were the most prevalent serovars (respectively 4/178 and 5/178) in a serosurvey on beef, sheep and deer farmers [159]. The risk factors for *Leptospira* seropositivity (all serovars combined) that the authors identified were: assisting in calving or fawning, a high abundance of wild deer on the farm, farming deer in combination with sheep and beef or alone, a proportion of flat terrain ≥ 25%, and a low abundance of possums. The latter was interpreted as the result of a confounding variable, although it is known that rats are more abundant where possums are not [168]. The abundance of rodents and hedgehogs on the farm (as estimated by the farmers) was not identified as a significant risk factor.

The recreational risk of contracting leptospirosis, especially around water-related activities, has been described in numerous developed countries [169,170,171], but no specific study has been conducted on that aspect in NZ. Nevertheless, one of the most common destinations (after Asia) where Australian travellers acquired leptospirosis was in NZ [172].

## 5. Discussion

Leptospirosis due to *L borgpetersenii* sv Ballum is emerging as an important problem in humans in NZ [19,20,173], but has not been thoroughly studied, and has even been overlooked in NZ in the past decades. There is a lack of current information on wildlife infection with Ballum in NZ, as most of the studies published were conducted almost 40 years ago. Mice are the main maintenance host of this serovar, and in NZ other species like hedgehogs, ship rats and brown rats are also able to maintain Ballum. It is interesting to note that, apart from a single isolation in Israel [41], NZ is the only country where descriptions of Ballum isolated from hedgehogs have been published. This review also highlighted the possibility for Ballum to spill over to livestock.

Other species of introduced mammals present in NZ were excluded from this review, but have rarely been studied (Appendix A and Supplementary Materials S1), and could also play a role. Feral pigs have been shown to harbour Ballum in other countries, but only one unpublished serosurvey was conducted in NZ almost 40 years ago, and all 60 feral pigs sampled were negative [111]. Very few lagomorphs have been tested in NZ [52,57], but Ballum was isolated on one occasion from a rabbit [52]. Serosurveys on wild ruminants did not include Ballum in their MAT panels [45,46,101], but recent serosurveys on farmed deer showed that Ballum could infect those species too [147].

The densities of the maintenance hosts, and more precisely the density of infected animals is an important parameter to understand the exposure to a pathogen. Mice, rats and hedgehogs are known to share the same habitat as humans and cattle, but there is limited information on their population density, demography and dynamics in NZ farm habitats.

Vaccination can theoretically lead to a shift in the predominant serovars found in a population. Given the uptake of vaccination against Pomona and Hardjobovis in dairy cattle, the ecological niche hypothesis may not be valid anymore for Ballum. The seroprevalence for Ballum apparently increased over time not only in dairy but also in beef cattle [147,157]. Cattle are able to harbour and shed this serovar, but the rate of wildlife-to-cattle transmission is unknown. Cattle could be becoming part of the maintenance community of Ballum. Recent information on what serovars are circulating in wildlife and vaccinated or unvaccinated sympatric livestock stratified by age would be needed.

Once infected, mice can excrete Ballum for the remainder of their lives, but this is not certain for rats and hedgehogs. Again, questions arise as to how the dynamics of mouse populations impact shedding over space and time. The role that the environmental reservoir plays in the survival and transmission of the pathogen to non-maintenance hosts also remains to be investigated. Leptospires of the species *L. borgpetersenii* are considered to be genetically disadvantaged for long survival in the environment, but nothing is known on that aspect specifically for Ballum.

Humans can be infected via different transmission pathways (Figure 1), but the relative importance of each transmission direction is currently unknown. The genetic, physiological and immunological attributes of humans as spillover hosts are also important determinants of transmission [22] that were beyond the scope of this work, but would also need to be considered. Do humans become contaminated mainly via contact with the maintenance hosts, bridge hosts or the environment? The lower incidence of Ballum in meat workers compared to stock or dairy farmers [20] indicates that direct contact with livestock is likely not the main route. Recent advances in molecular methods and genotyping to discriminate among those different sources could be a way to assess this. It emerges from descriptive studies of human notified cases that the risk factors for Ballum differ from Hardjobovis and Pomona, indicating likely different transmission pathways. Understanding the diversity and relative importance of different sources of infection will be critical for the efficient control of leptospirosis.

In conclusion, most of the knowledge available for Ballum in NZ relies on studies performed in the 1970s in both domestic and wild animals. After a long gap with no available information on this serovar, recent investigations of livestock and human epidemiological data indicated that more information is needed about the role of wild hosts in the maintenance and transmission of Ballum. The possibility for this serovar to spill over to domestic hosts, which could thus act as bridge hosts, should also be considered.

## Supplementary Materials

The following are available online at https://www.mdpi.com/article/10.3390/tropicalmed6040189/s1. Supplementary Materials S1: Results of the preliminary literature search on wildlife as a source of *Leptospira* infection in NZ.

## Appendix A. Preliminary Literature Search on Wildlife as a Source of *Leptospira* Infection in NZ

### Methodology

The preliminary literature search algorithm was #Leptospirosis AND #NZ AND #Wildlife. The keywords and search strings used in Web of Science are detailed in Table A1. The online databases used were Scopus, Web of Science, and SciQuest. The searches were also performed on the references cited in the articles retrieved. Google Scholar, the Massey University library database (Discover) and NZResearch.org.nz were searched for additional grey literature on *Leptospira* in NZ, as well as the paper archives of the Leptospirosis Reference Centre in Amsterdam (spanning 1915–1990), which were searched for the keywords ‘New Zealand’, ‘hedgehog’ and ‘Ballum’. The searches were conducted between January 2018 and September 2018.

**Table A1:** Electronic search strategy used for Web Of Science, with detail on the search strings used (#Leptospirosis AND #NZ AND #Wildlife) to identify the wild maintenance hosts of *Leptospira borgpetersenii* serovar Ballum in New Zealand to include in the literature review.

Terms	Search Strings
#Leptospirosis	Leptospir* OR “Weil disease” OR “Weil’s disease” OR “dairy farm fever” NOT Leptospirill*
#NZ	New-Zealand OR “New Zealand” OR Aotearoa
#Wildlife	#Wild OR #Hedgehog OR #Rodents OR #Mustelid OR #Ruminants OR #Wildboar OR #Possum
#Wild	Wild OR wildlife OR free-ranging OR “free ranging” OR feral
#Hedgehog	hedgehog OR “*Erinaceus europaeus*”
#Rodents	rodent OR rodents OR rat OR rats OR *Rattus* OR mice OR “*Mus musculus*” OR mouse OR murine OR kiore
#Ruminants	deer OR *Cervus* OR *Axis* OR *Alces* OR moose OR chamois OR *Rupicapra* OR *Odocoileus* OR *Dama* OR Tahr OR *Hemitragus* OR “feral goat” OR “feral sheep”
#Mustelid	*Mustela* OR stoat* OR ferret* OR weasel*
#Possum	possum OR *Trichosurus*
#Wildboar	(“*Sus scrofa*” AND (wild OR feral)) OR”wild pig” OR “feral pig” OR “wild boar”

Known and suspected wild maintenance hosts of *Leptospira* spp. in NZ.

Nearly all species of wild terrestrial mammals introduced to NZ have previously been investigated for exposure to *Leptospira* spp. As a result, only the brush-tailed possum was considered to be of interest as a maintenance host of *L. borgpetersenii* sv Balcanica, and rats, mice and hedgehogs were considered to be of interest as maintenance hosts of Ballum.

Other NZ wild species have typically been considered as insignificant hosts for the maintenance and propagation of domestic animal or human leptospirosis, and have rarely been studied. The studied species for which an absence of serological titres for *Leptospira* spp. has been reported in NZ include feral pig (*Sus scrofa*) [111], hare (*Lepus europaeus*), stoat (*Mustela erminea*), ferret (*Mustela putorious furo*), weasel (*Mustela nivalis*) [15], red deer (*Cervus elaphus*) [57], sika deer (*Cervus nippon*), fallow deer (*Dama dama*), white-tailed deer (*Odocoileus virginianus borealis*), chamois (*Rupicapra rupicapra*), and Himalayan tahr (*Hemitragus jemlahicus*) [46,57].

On occasion, MAT titres were found in wallabies (probably dama wallabies, *Macropus eugenii*) against Hardjobovis and Ballum [111]; in feral cats (*Felis catus*) against Pomona and Ballum [15]; in feral goats (*Capra hircus*) against Pomona, Hardjobovis, Balcanica and Ballum [110]; in hunted deer (*Cervus elaphus*) against Pomona and Copenhageni [45,101,174]; and more recently in fur seal (*Arctocephalus forsteri*) against Canicola, Hardjobovis and Pomona, and in sea lions (*Phocarctos hookeri*) against Pomona [107,109]. Despite many low sample sizes, these results were interpreted as sporadic infections through contact with known maintenance species of these serovars (livestock, rodents or other wild species).

The literature available is synthesized in the Supplementary Materials S1. All of the publications relied on serological methods (agglutination, CF, MAT), and only some on bacterial cultures from blood, urine or kidney, and subsequent typing by serotyping, a cross-agglutination absorption test or a restriction-endonuclease analysis. Some studies assumed that leptospires isolated or observed under DFM belonged to a certain serovar without typing them, e.g., [42,44]. Importantly, the number of animals tested was limited for several studies. Furthermore, Ballum was not included in the test panel in numerous studies (shaded in the Supplementary Materials S1), and few studies were conducted outside of the North Island. Given that some wild or feral species were not tested, or only tested in limited numbers, it is possible that carriage of Ballum (or other serovars) may have been missed.

The recognised maintenance hosts of Ballum present in NZ targeted in this article are: the mouse, ship rat, brown rat and hedgehog.
